# Integrin β1 signaling at the crossroads of cellular plasticity, the tumor microenvironment, and therapy resistance in medulloblastoma

**DOI:** 10.3389/fonc.2026.1930948

**Published:** 2026-08-11

**Authors:** William Echavidre, Vincent Picco, Christopher Montemagno

**Affiliations:** Medical Biology Department, Centre Scientifique de Monaco, Monaco, Monaco

**Keywords:** cancer stem cells, extracellular matrix, integrin β1, medulloblastoma, microenvironment, pediatric oncology, radioresistance

## Abstract

Medulloblastoma (MB), the most common malignant pediatric brain tumor, remains associated with relapse and long-term treatment-related toxicities. Tumor–microenvironment interactions increasingly appear to contribute to progression, stemness, and therapy resistance in brain cancers. Integrin β1, a major mediator of cell–extracellular matrix interactions, regulates survival signaling, mechanotransduction, and cancer stem cell maintenance in several tumor types, particularly glioblastoma. This mini-review discusses how developmental programs, extracellular matrix remodeling, hypoxia, and cancer stem cell plasticity may converge through integrin-dependent signaling in MB. Although direct evidence remains limited, converging findings from developmental neurobiology, stem cell biology, and cancer research support integrin β1 as an underexplored candidate regulator of MB progression and therapeutic resistance.

## 1 Introduction

Medulloblastoma (MB) is the most common malignant pediatric brain tumor and remains associated with significant long-term morbidity and mortality despite advances in multimodal therapies combining surgery, radiotherapy, and chemotherapy (1–3). Survivors frequently experience severe neurocognitive, endocrine, and developmental impairments resulting from treatment-related toxicities (4, 5). Relapse remains frequent and is associated with poor prognosis and limited therapeutic options (6, 7).

Large-scale genomic and epigenomic studies have redefined MB as a heterogeneous disease composed of four principal molecular subgroups—WNT, SHH, Group 3, and Group 4—each characterized by distinct developmental origins, molecular alterations, and clinical outcomes (8–10). While WNT tumors are generally associated with favorable prognosis, Group 3 tumors display the poorest prognosis. Despite these advances in molecular classification, current therapeutic strategies remain relatively similar across subgroups, highlighting the need for biologically informed therapeutic approaches (11).

Increasing evidence indicates that tumor progression and therapy resistance are not solely driven by tumor-intrinsic alterations but are also strongly influenced by interactions with the tumor microenvironment (TME) (12, 13). The extracellular matrix (ECM), a major component of the TME, regulates proliferation, migration, survival, and cellular plasticity through biochemical and mechanical signaling. In brain tumors, ECM remodeling and microenvironmental signaling have emerged as important regulators of stemness and therapeutic response (14, 15).

Integrins are transmembrane receptors that mediate cell–ECM interactions and transduce extracellular signals into intracellular signaling pathways (15, 16). Among them, integrin β1 is of particular interest because of its ability to form multiple heterodimers and interact with diverse ECM ligands, including laminins, fibronectin, and collagens (17, 18). Through activation of FAK, PI3K/AKT, and MAPK pathways, integrin β1 regulates survival, migration, mechanotransduction, and stem-like behavior (19, 20).

In several cancers, particularly glioblastoma, integrin β1 signaling has been implicated in cancer stem cell maintenance, invasion, and resistance to therapy (20–22). However, despite the growing recognition of microenvironmental regulation in MB biology, the role of integrin β1 in this disease remains poorly characterized.

Recent reviews have highlighted the importance of the tumor microenvironment in MB biology (23), while integrin signaling and cellular plasticity have been extensively reviewed in the broader context of cancer (17, 20, 21). However, these concepts have rarely been integrated into a unified framework in medulloblastoma. In this mini-review, we discuss how developmental signaling, extracellular matrix interactions, and tumor cell plasticity may converge through integrin β1-dependent pathways in MB. By considering integrin β1 as a potential molecular link between these processes, we explore the implications of this integrative framework for therapy resistance and future therapeutic strategies.

## 2 Developmental and microenvironmental context of medulloblastoma

MB is widely regarded as a developmental tumor arising from dysregulation of cerebellar ontogeny (24, 25). Cerebellar development depends on coordinated proliferation, migration, and differentiation of neural progenitor populations controlled by SHH and WNT signaling. Granule cell progenitors (GCPs) within the external granular layer are considered a major cell population involved in MB tumorigenesis (26–28). SHH signaling secreted by Purkinje cells promotes GCP proliferation during normal development, whereas aberrant activation of this pathway through mutations in PTCH1 or SMO drives uncontrolled proliferation in SHH-subgroup MB. Similarly, activation of the WNT pathway through CTNNB1 mutations characterizes the WNT subgroup and is associated with favorable prognosis (29, 30).

Unlike many adult cancers, MB exhibits a relatively low mutational burden and appears to rely more heavily on epigenetic dysregulation and persistence of developmental programs (31–33). This supports the idea that MB arises from hijacking developmental programs rather than from progressive accumulation of genomic alterations.

Importantly, cerebellar development is strongly influenced by interactions with the extracellular matrix (ECM) (34–37). ECM components such as laminins, fibronectin, and collagens regulate progenitor adhesion, migration, and differentiation through integrin-mediated signaling (34, 36). Among integrins, β1-containing integrins play essential roles in neural development by regulating progenitor adhesion, survival, and tissue organization (38–40). Experimental disruption of integrin β1 signaling leads to defects in brain architecture and abnormal cell positioning, highlighting its importance in neural morphogenesis.

Moreover, the contribution of integrin β1 is likely to vary across MB molecular subgroups. Single-cell transcriptomic studies have shown that WNT, SHH, Group 3, and Group 4 tumors contain distinct malignant cell states that reflect different developmental lineages and differentiation programs (31, 33). In addition, subgroup-specific differences have been reported in the composition of the immune and stromal microenvironment (41, 42). Group 3 and Group 4 tumors should not be regarded as entirely discrete biological entities, as transcriptomic analyses indicate that they form a biological continuum containing multiple intermediate states (43). These differences in cellular identity and microenvironmental organization may influence ECM composition and consequently β1-dependent signaling. However, whether specific MB subgroups or cellular states exhibit preferential dependence on integrin β1 remains to be directly established, representing an important area for future investigation.

These differences in cellular identity and microenvironmental organization may influence ECM composition and consequently β1-dependent signaling. Single-cell transcriptomic studies have refined our understanding of MB cellular heterogeneity; however, the functional contribution of integrin β1 across these transcriptionally defined cellular states remains poorly characterized.

Collectively, these observations suggest that integrin-mediated signaling may contribute not only to normal cerebellar development but also to tumor initiation and progression in MB. Although direct evidence remains limited, the developmental dependence of MB on ECM-regulated signaling pathways provides a strong rationale for investigating integrin β1 as a regulator of tumor cell identity and microenvironmental interactions.

## 3 Cancer stem cell plasticity and ECM niches in medulloblastoma

Tumor heterogeneity in medulloblastoma is increasingly understood as a dynamic process driven by cancer stem cell (CSC) plasticity and microenvironmental regulation (44–47). CSCs were initially described as tumor-propagating cells capable of self-renewal and long-term tumor maintenance (48, 49). These populations are now recognized as major contributors to relapse, therapeutic resistance, and tumor adaptation (50).

However, the classical hierarchical CSC model has progressively evolved toward a more dynamic view in which tumor cells transition between stem-like and differentiated states in response to intrinsic transcriptional programs and extrinsic environmental cues (46, 47). In brain tumors, including MB, this plasticity appears particularly relevant because tumor cells retain features of developmental lineages and progenitor programs.

Single-cell transcriptomic studies further support this model by showing that MB cells occupy multiple differentiation states reflecting developmental trajectories rather than rigid hierarchies (31, 33). These studies reveal that tumor cells retain considerable phenotypic plasticity and can occupy distinct transcriptional states associated with neural progenitor differentiation programs. Together, these findings support a dynamic continuum of tumor cell states shaped by both intrinsic developmental programs and extrinsic microenvironmental cues. Although direct evidence linking integrin β1 to these transcriptionally defined cell states remains limited, its established role in ECM sensing, mechanotransduction, and stem cell regulation suggests that it may contribute to the maintenance and adaptation of these plastic cell populations.

Hypoxia also promotes stem-like phenotypes, metabolic adaptation, and therapeutic resistance through HIF signaling, and has been associated with aggressive behavior in MB (51–53). The ECM constitutes a major functional component of the TME and strongly influences CSC behavior (14, 54). Laminin-rich environments promote neural stem cell maintenance and proliferation, whereas fibronectin-rich matrices are more frequently associated with migration and invasion (55–57). These effects are mediated largely through β1-containing integrins, which connect ECM-derived signals to intracellular pathways regulating adhesion, survival, and self-renewal (16, 18, 58).

In addition to regulating stem cell behavior, integrin β1 may also contribute to the invasive properties of MB. Unlike gliomas, which primarily invade the surrounding brain parenchyma, MB characteristically disseminates through the cerebrospinal fluid and colonizes the leptomeninges and is associated with poor clinical outcome. Experimental studies have demonstrated that β1-containing integrins, particularly the α9β1 heterodimer, mediate MB cell adhesion to tenascin-C, an extracellular matrix glycoprotein enriched within the leptomeningeal environment (59). This interaction promotes activation of signaling pathways involved in cell survival and proliferation, suggesting that integrin β1-dependent adhesion may facilitate the establishment and persistence of disseminated tumor cells. Although the precise contribution of β1 signaling to leptomeningeal colonization remains poorly understood, its central role in ECM recognition and cell–matrix interactions provides a compelling rationale for further investigation.

In glioblastoma, integrin β1 signaling promotes CSC maintenance and tumor initiation through activation of focal adhesion kinase (FAK) and PI3K/AKT pathways (20, 21). Integrin β1 also contributes to invasion and interactions with vascular niches, reinforcing its role as a central regulator of tumor progression (21, 60, 61). Beyond biochemical signaling, integrin β1 mediates mechanotransduction, allowing tumor cells to sense ECM stiffness and architecture (62, 63). Increased matrix stiffness has been associated with enhanced stemness and aggressive behavior in multiple cancers (63, 64).

Importantly, integrin β1 signaling intersects with developmental pathways central to MB biology, including SHH and WNT signaling. Crosstalk between integrins and receptor tyrosine kinases further amplifies downstream signaling networks involved in survival and proliferation (17, 65). Together, these observations support the hypothesis that integrin β1 may function as an integrative hub coordinating developmental and microenvironmental signals in MB.

Although direct evidence specifically implicating integrin β1 in MB CSC regulation remains limited, the strong parallels with other brain tumors provide a compelling rationale for further investigation.

## 4 Integrin β1 and radioresistance in medulloblastoma

Radiotherapy remains a cornerstone of medulloblastoma treatment and is a major determinant of patient outcome (2, 3). Nevertheless, resistance to ionizing radiation remains a major clinical challenge, particularly in recurrent disease where prognosis is poor and therapeutic options are limited (6, 7).

Radioresistance results from tumor-intrinsic and microenvironment-dependent mechanisms, including efficient DNA damage repair, redox control, hypoxia, and therapy-induced plasticity (22, 53, 66, 67). CSC-like populations often display enhanced DNA repair, reduced ROS levels, and lower sensitivity to genotoxic stress (68, 69). Hypoxia further limits radiation-induced DNA damage fixation while promoting stem-like phenotypes through HIF signaling (51, 53, 70). Together, these processes may contribute to MB relapse, although direct evidence remains limited.

Beyond tumor-intrinsic mechanisms, the tumor microenvironment plays a central role in regulating radiation response (12, 13, 22). Adhesion to ECM components activates integrin-mediated signaling pathways that enhance survival and resistance to apoptosis following irradiation (22, 71). This phenomenon, referred to as cell adhesion-mediated radioresistance (CAM-RR), has been described in several tumor types.

Integrin β1 appears to be a major mediator of CAM-RR through activation of signaling pathways such as FAK, PI3K/AKT, and MAPK (19, 22). Inhibition of integrin β1 enhances radiosensitivity in several cancer models by increasing apoptosis and impairing DNA repair (71, 72). In glioblastoma, ECM-dependent integrin signaling contributes to resistance through interactions with laminin- and fibronectin-rich microenvironments (21, 44).

Integrin signaling may also modulate chromatin organization and accessibility of DNA repair machinery (73). In parallel, radiation-induced ECM remodeling, including increased collagen and fibronectin deposition, may further reinforce integrin signaling and establish a protective niche favoring tumor survival (74, 75).

**Figure 1:**
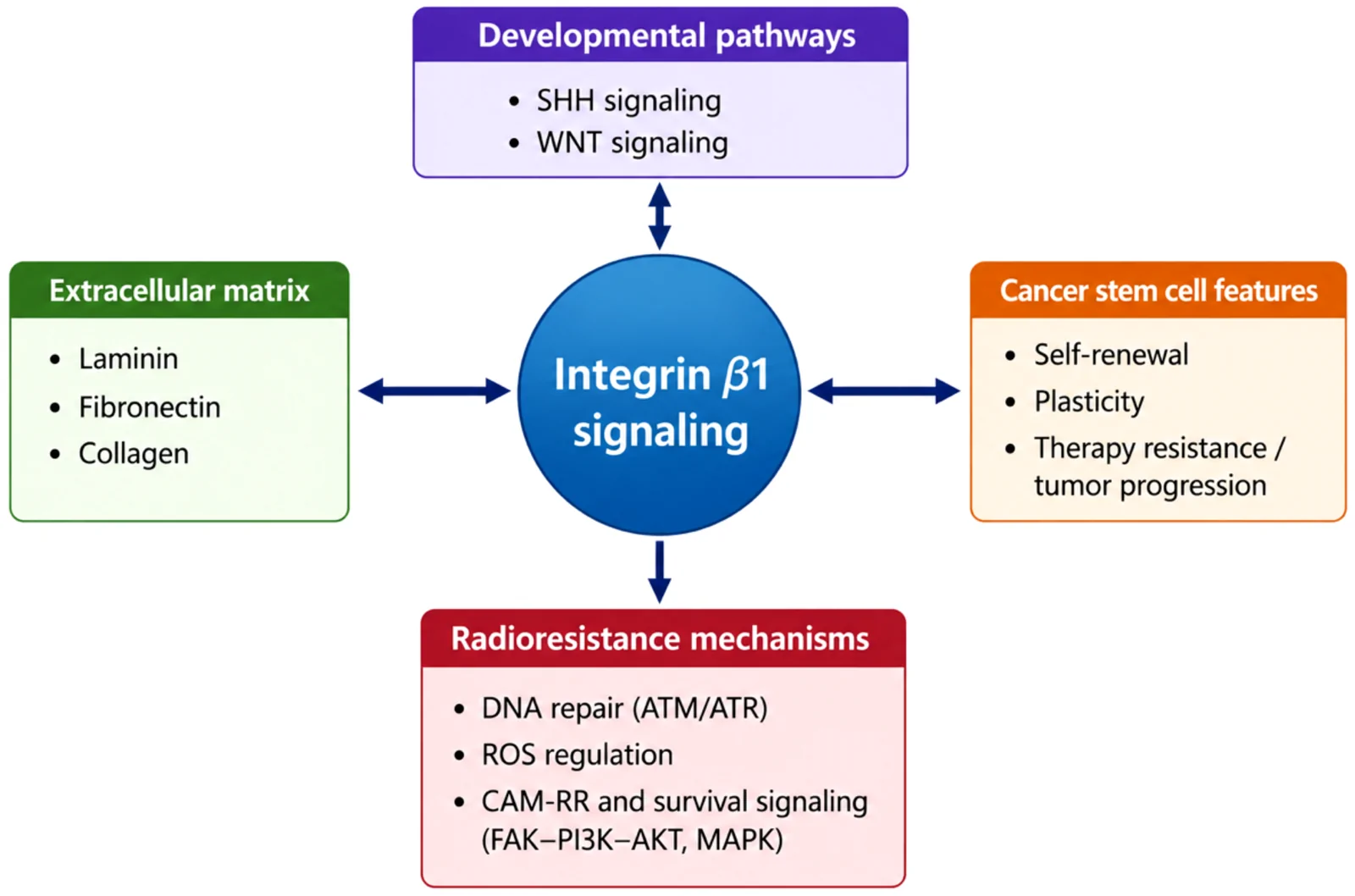
Integrin β1 as a central hub linking developmental signaling, extracellular matrix interactions, stemness, and radioresistance in medulloblastoma. Integrin β1 signaling integrates extracellular matrix (ECM)-derived cues, including laminin, fibronectin, and collagen interactions, with developmental pathways such as SHH and WNT. Through activation of downstream signaling pathways including FAK, PI3K/AKT, and MAPK, β1 integrin may regulate cancer stem cell features, cellular plasticity, tumor progression, and mechanisms associated with radioresistance, including DNA repair, ROS regulation, and survival signaling.

Collectively, these observations position integrin β1 as a potential regulator linking ECM interactions, CSC plasticity, developmental signaling, and radioresistance in MB (**Figure 1**). Although direct experimental evidence remains scarce, the strong biological convergence observed across developmental biology, stem cell regulation, and radiobiology supports further investigation of this pathway in pediatric brain tumors.

## 5 Integrin β1 as a signaling and mechanotransduction hub

Integrin β1 forms heterodimeric receptors with multiple α subunits, enabling interactions with ECM ligands such as laminins, fibronectin, and collagens (16, 18). Upon ligand engagement, integrin β1 promotes focal adhesion assembly and activates FAK/Src signaling, which integrates biochemical and mechanical signals originating from the extracellular matrix. Through downstream PI3K/AKT, MAPK/ERK, and Rho GTPase pathways, integrin β1 regulates cell survival, proliferation, migration, cytoskeletal remodeling, and mechanotransduction (19, 20). Mechanical forces transmitted through integrin β1-dependent adhesions can regulate the activity and nuclear localization of the mechanosensitive transcriptional co-activators YAP and TAZ, thereby coupling extracellular matrix stiffness to transcriptional programs involved in proliferation, survival, and cellular plasticity (76, 77).

**Figure 2:**
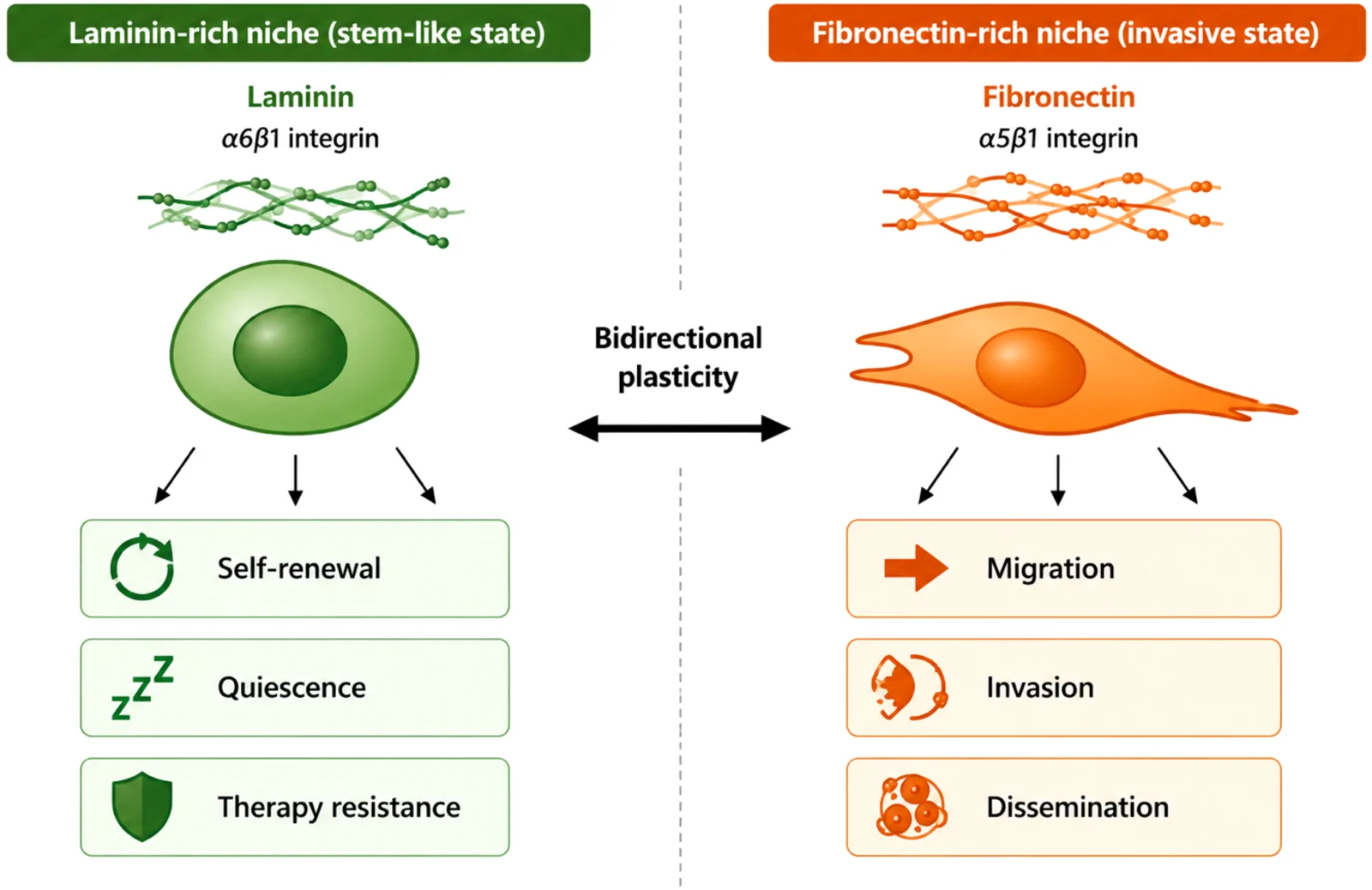
Distinct extracellular matrix niches may regulate tumor cell states through β1 integrin signaling in medulloblastoma. Laminin-rich microenvironments interacting predominantly with α6β1 integrin are associated with stem-like properties, including self-renewal, quiescence, and therapy resistance. In contrast, fibronectin-rich niches engaging α5β1 integrin are linked to migratory and invasive phenotypes associated with tumor dissemination. Bidirectional transitions between these cellular states illustrate tumor plasticity driven by extracellular matrix composition and microenvironmental signaling.

Importantly, the functional consequences of integrin β1 signaling depend strongly on its α-subunit partner and microenvironmental context. For example, α6β1 integrin preferentially interacts with laminin-rich niches associated with stem cell maintenance, whereas α5β1 integrin primarily binds fibronectin-rich matrices linked to migration and invasion (44, 55, 78). This functional diversity enables integrin β1 to regulate distinct tumor cell states depending on ECM composition (**Figure 2**).

Integrin β1 signaling also displays extensive crosstalk with receptor tyrosine kinases and developmental signaling pathways (17, 65). Interactions with EGFR, SHH, and WNT pathways may amplify survival signaling and influence tumor cell plasticity (17, 20). Such integrative signaling networks suggest that integrin β1 functions not merely as an adhesion receptor but rather as a central signaling hub coordinating biochemical and mechanical inputs from the tumor microenvironment.

Beyond its interactions with the extracellular matrix, the MB tumor microenvironment also comprises immune cell populations, including resident microglia, infiltrating macrophages, and, to a lesser extent, lymphocytes, which may influence tumor progression and therapeutic response. The composition and functional organization of this immune microenvironment vary across MB molecular subgroups (41, 42). More broadly, the ECM contributes to bidirectional communication between tumor cells, stromal cells, and immune populations by regulating cell adhesion, cytokine availability, and tissue remodeling (14). In other solid tumors, integrin-dependent signaling contributes to bidirectional communication between tumor and immune cells by modulating cytokine signaling, immune cell recruitment, and macrophage polarization, thereby promoting an immunosuppressive tumor microenvironment (16, 79). Although the specific contribution of integrin β1 to immune regulation in MB remains poorly understood, its central role in cell–matrix interactions and bidirectional communication between tumor cells and the surrounding microenvironment suggests that it may influence immune cell recruitment, localization, or functional polarization within the tumor microenvironment.

Although these mechanisms have been extensively characterized in other tumor types, their relevance in MB remains insufficiently explored. Given the developmental origin of MB and the importance of ECM-dependent signaling in cerebellar biology, integrin β1 may represent a critical regulator of tumor adaptation and progression (24, 34, 38).

## 6 Therapeutic opportunities and translational challenges

Given its central role in stemness, survival signaling, and microenvironmental interactions, integrin β1 represents an attractive therapeutic target in cancer. Preclinical studies in multiple tumor types have demonstrated that inhibition of integrin β1 signaling can enhance sensitivity to chemotherapy and radiotherapy by disrupting survival pathways and impairing ECM-mediated protection (22, 71, 72, 80).

Several therapeutic approaches targeting integrin β1 signaling have been explored, including β1-blocking monoclonal antibodies such as AIIB2 (Park et al., 2006), peptide antagonists, and inhibitors of downstream signaling molecules such as focal adhesion kinase (FAK) (17, 81, 82). Preclinical studies using β1-blocking antibodies have demonstrated reduced tumor growth and enhanced sensitivity to radiotherapy in several cancer models (72, 81), whereas clinically investigated FAK inhibitors, including defactinib and GSK2256098, represent complementary strategies to disrupt integrin-dependent signaling (82, 83). Although β1-targeting antibodies have shown encouraging activity in preclinical models and FAK inhibitors have advanced into early-phase clinical evaluation, none has yet been specifically evaluated in MB.

However, clinical translation of integrin-targeting therapies has proven challenging. Cilengitide, a cyclic RGD peptide developed primarily to target αvβ3 and αvβ5 integrins (84), failed to improve overall survival when combined with standard radiotherapy and temozolomide in patients with newly diagnosed glioblastoma in the phase III CENTRIC trial (85). Importantly, this lack of clinical benefit should not be interpreted as evidence against integrin β1-directed therapeutic strategies, as cilengitide was not developed as a selective β1 inhibitor and integrin signaling is highly dependent on receptor subtype, cellular context, ligand availability, and compensatory signaling among different integrin heterodimers (17, 20, 86).

Another major challenge is tumor heterogeneity. Integrin expression and activity vary between tumor subtypes and even between distinct cellular states within the same tumor (10, 86). This suggests that effective therapeutic targeting will likely require improved patient stratification and identification of biomarkers predicting integrin dependency.

In MB, β1 targeting remains largely unexplored, but developmental signaling, CSC plasticity, ECM-mediated resistance, and the central role of radiotherapy support its investigation (20–22). Future therapeutic approaches will likely require rational combination strategies rather than integrin inhibition alone. Combining integrin β1 targeting with radiotherapy could simultaneously enhance DNA damage while blocking ECM-mediated survival signaling (22, 71). Similarly, combining integrin inhibition with SHH or WNT pathway inhibitors may improve efficacy by targeting both tumor-intrinsic and microenvironmental mechanisms.

Beyond direct β1 inhibition, targeting downstream effectors such as FAK may represent an alternative therapeutic strategy capable of overcoming compensatory signaling and some limitations associated with integrin-directed therapies (82, 83). In parallel, advances in molecular profiling and spatial transcriptomics may facilitate identification of MB subgroups or microenvironmental contexts particularly dependent on integrin signaling (31, 33).

Overall, despite significant translational challenges, integrin β1 remains a promising candidate for therapeutic investigation in MB, particularly in the context of combination therapies aimed at overcoming stemness-associated resistance mechanisms.

## 7 Future perspectives and conclusions

Increasing evidence indicates that medulloblastoma progression and therapeutic resistance are shaped not only by tumor-intrinsic genetic alterations but also by dynamic interactions with the tumor microenvironment (12, 13, 20). Within this framework, integrin β1 emerges as a potentially important regulator linking extracellular matrix signaling, stemness, cellular plasticity, and resistance to therapy.

Although direct evidence specifically implicating integrin β1 in MB remains limited, strong biological parallels with neural development, glioblastoma, and other cancers support the hypothesis that this pathway may play an important role in disease progression (20, 38, 44). The convergence of developmental signaling, ECM remodeling, mechanotransduction, and CSC regulation around integrin β1 suggests that it may function as a central integrative hub in MB biology (63, 74).

Future studies should aim to characterize integrin β1 expression and activity across MB molecular subgroups and determine whether specific tumor contexts exhibit increased dependency on ECM-mediated signaling. Emerging technologies such as spatial transcriptomics, single-cell multiomics, organoid models, and engineered ECM systems may provide new opportunities to investigate these mechanisms in physiologically relevant settings.

Particular attention should also be given to therapy-induced microenvironmental remodeling (75). Radiotherapy may alter ECM composition and mechanical properties in ways that reinforce integrin signaling and promote tumor adaptation. Understanding these dynamic interactions could help identify new therapeutic vulnerabilities.

Ultimately, integrating microenvironmental biology into the study of medulloblastoma may help bridge the gap between molecular classification and therapeutic innovation. In this context, integrin β1 represents a compelling but underexplored candidate deserving further investigation in pediatric neuro-oncology.

## References

[1] Northcott PA Dubuc AM Pfister S Taylor MD . Molecular subgroups of medulloblastoma. Expert Rev Neurother. (2012) 12:871–84. doi: 10.1586/ern.12.66 22853794 PMC4334443

[2] Robinson GW Rudneva VA Buchhalter I Billups CA Waszak SM Smith KS . Risk-adapted therapy for young children with medulloblastoma (SJYC07): therapeutic and molecular outcomes from a multicentre, phase 2 trial. Lancet Oncol. (2018) 19:768–84. doi: 10.1016/S1470-2045(18)30204-3 29778738 PMC6078206

[3] Gajjar A Robinson GW Smith KS Lin T Merchant TE Chintagumpala M . Outcomes by clinical and molecular features in children with medulloblastoma treated with risk-adapted therapy: Results of an international phase III trial (SJMB03). J Clin Oncol. (2021) 39:822–35. doi: 10.1200/JCO.20.01372 33405951 PMC10166353

[4] Stavinoha PL Askins MA Powell SK Pillay Smiley N Robert RS . Neurocognitive and psychosocial outcomes in pediatric brain tumor survivors. Bioengineering (Basel). (2018) 5:73. doi: 10.3390/bioengineering5030073 30208602 PMC6164803

[5] Robison LL Hudson MM . Survivors of childhood and adolescent cancer: life-long risks and responsibilities. Nat Rev Cancer. (2014) 14:61–70. doi: 10.1038/nrc3634 24304873 PMC6425479

[6] Hill RM Richardson S Schwalbe EC Hicks D Lindsey JC Crosier S . Time, pattern, and outcome of medulloblastoma relapse and their association with tumour biology at diagnosis and therapy: a multicentre cohort study. Lancet Child Adolesc Health. (2020) 4:865–74. doi: 10.1016/S2352-4642(20)30246-7 33222802 PMC7671998

[7] Ramaswamy V Remke M Bouffet E Faria CC Perreault S Cho Y-J . Recurrence patterns across medulloblastoma subgroups: an integrated clinical and molecular analysis. Lancet Oncol. (2013) 14:1200–7. doi: 10.1016/S1470-2045(13)70449-2 24140199 PMC3953419

[8] Taylor MD Northcott PA Korshunov A Remke M Cho Y-J Clifford SC . Molecular subgroups of medulloblastoma: the current consensus. Acta Neuropathol. (2012) 123:465–72. doi: 10.1007/s00401-011-0922-z 22134537 PMC3306779

[9] Northcott PA Korshunov A Witt H Hielscher T Eberhart CG Mack S . Medulloblastoma comprises four distinct molecular variants. J Clin Oncol. (2011) 29:1408–14. doi: 10.1200/JCO.2009.27.4324 20823417 PMC4874239

[10] Cavalli FMG Remke M Rampasek L Peacock J Shih DJH Luu B . Intertumoral heterogeneity within medulloblastoma subgroups. Cancer Cell. (2017) 31:737–754.e6. doi: 10.1016/j.ccell.2017.05.005 28609654 PMC6163053

[11] Juraschka K Taylor MD . Medulloblastoma in the age of molecular subgroups: a review: JNSPG 75th anniversary invited review article. J Neurosurgery: Pediatr. (2019) 24:353–63. doi: 10.3171/2019.5.PEDS18381 31574483

[12] Junttila MR de Sauvage FJ . Influence of tumour micro-environment heterogeneity on therapeutic response. Nature. (2013) 501:346–54. doi: 10.1038/nature12626 24048067

[13] Quail DF Joyce JA . The microenvironmental landscape of brain tumors. Cancer Cell. (2017) 31:326–41. doi: 10.1016/j.ccell.2017.02.009 28292436 PMC5424263

[14] Pickup MW Mouw JK Weaver VM . The extracellular matrix modulates the hallmarks of cancer. EMBO Rep. (2014) 15:1243–53. doi: 10.15252/embr.201439246 25381661 PMC4264927

[15] Hynes RO . The extracellular matrix: not just pretty fibrils. Science. (2009) 326:1216–9. doi: 10.1126/science.1176009 19965464 PMC3536535

[16] Desgrosellier JS Cheresh DA . Integrins in cancer: biological implications and therapeutic opportunities. Nat Rev Cancer. (2010) 10:9–22. doi: 10.1038/nrc2748 20029421 PMC4383089

[17] Hamidi H Ivaska J . Every step of the way: integrins in cancer progression and metastasis. Nat Rev Cancer. (2018) 18:533–48. doi: 10.1038/s41568-018-0038-z 30002479 PMC6629548

[18] Humphries JD Chastney MR Askari JA Humphries MJ . Signal transduction via integrin adhesion complexes. Curr Opin Cell Biol. (2019) 56:14–21. doi: 10.1016/j.ceb.2018.08.004 30195153

[19] Mitra SK Hanson DA Schlaepfer DD . Focal adhesion kinase: in command and control of cell motility. Nat Rev Mol Cell Biol. (2005) 6:56–68. doi: 10.1038/nrm1549 15688067

[20] Cooper J Giancotti FG . Integrin signaling in cancer: mechanotransduction, stemness, epithelial plasticity, and therapeutic resistance. Cancer Cell. (2019) 35:347–67. doi: 10.1016/j.ccell.2019.01.007 30889378 PMC6684107

[21] Seguin L Desgrosellier JS Weis SM Cheresh DA . Integrins and cancer: regulators of cancer stemness, metastasis, and drug resistance. Trends Cell Biol. (2015) 25:234–40. doi: 10.1016/j.tcb.2014.12.006 25572304 PMC4380531

[22] Eke I Cordes N . Focal adhesion signaling and therapy resistance in cancer. Semin Cancer Biol. (2015) 31:65–75. doi: 10.1016/j.semcancer.2014.07.009 25117005

[23] van Bree NFHN Wilhelm M . The tumor microenvironment of medulloblastoma: an intricate multicellular network with therapeutic potential. Cancers (Basel). (2022) 14:5009. doi: 10.3390/cancers14205009 36291792 PMC9599673

[24] Pei Y Brun SN Markant SL Lento W Gibson P Taketo MM . WNT signaling increases proliferation and impairs differentiation of stem cells in the developing cerebellum. Development. (2012) 139:1724–33. doi: 10.1242/dev.050104 22461560 PMC3328175

[25] Wang W Shiraishi R Kawauchi D . Sonic hedgehog signaling in cerebellar development and cancer. Front Cell Dev Biol. (2022) 10. doi: 10.3389/fcell.2022.864035 35573667 PMC9100414

[26] Wallace VA . Purkinje-cell-derived Sonic hedgehog regulates granule neuron precursor cell proliferation in the developing mouse cerebellum. Curr Biol. (1999) 9:445–8. doi: 10.1016/s0960-9822(99)80195-x 10226030

[27] Wechsler-Reya RJ Scott MP . Control of neuronal precursor proliferation in the cerebellum by Sonic hedgehog. Neuron. (1999) 22:103–14. doi: 10.1016/s0896-6273(00)80682-0 10027293

[28] Goodrich LV Milenković L Higgins KM Scott MP . Altered neural cell fates and medulloblastoma in mouse patched mutants. Science. (1997) 277:1109–13. doi: 10.1126/science.277.5329.1109 9262482

[29] Ellison DW Onilude OE Lindsey JC Lusher ME Weston CL Taylor RE . Beta-catenin status predicts a favorable outcome in childhood medulloblastoma: the United Kingdom Children’s Cancer Study Group Brain Tumour Committee. J Clin Oncol. (2005) 23:7951–79. doi: 10.1200/JCO.2005.01.5479 16258095

[30] Eberhart CG Tihan T Burger PC . Nuclear localization and mutation of beta-catenin in medulloblastomas. J Neuropathol Exp Neurol. (2000) 59:333–7. doi: 10.1093/jnen/59.4.333 10759189

[31] Hovestadt V Smith KS Bihannic L Filbin MG Shaw ML Baumgartner A . Resolving medulloblastoma cellular architecture by single-cell genomics. Nature. (2019) 572:74–9. doi: 10.1038/s41586-019-1434-6 31341285 PMC6754173

[32] Gröbner SN Worst BC Weischenfeldt J Buchhalter I Kleinheinz K Rudneva VA . The landscape of genomic alterations across childhood cancers. Nature. (2018) 555:321–7. doi: 10.1038/nature25480 29489754

[33] Vladoiu MC El-Hamamy I Donovan LK Farooq H Holgado BL Sundaravadanam Y . Childhood cerebellar tumours mirror conserved fetal transcriptional programs. Nature. (2019) 572:67–73. doi: 10.1038/s41586-019-1158-7 31043743 PMC6675628

[34] Long KR Huttner WB . How the extracellular matrix shapes neural development. Open Biol. (2019) 9:180216. doi: 10.1098/rsob.180216 30958121 PMC6367132

[35] Frantz C Stewart KM Weaver VM . The extracellular matrix at a glance. J Cell Sci. (2010) 123:4195–200. doi: 10.1242/jcs.023820 21123617 PMC2995612

[36] Barros CS Franco SJ Müller U . Extracellular matrix: functions in the nervous system. Cold Spring Harb Perspect Biol. (2011) 3:a005108. doi: 10.1101/cshperspect.a005108 21123393 PMC3003458

[37] Heng C Lefebvre O Klein A Edwards M Simon-Assmann P Orend G . Functional role of laminin α1 chain during cerebellum development. Cell Adhesion Migration. (2011) 5:480–9. doi: 10.4161/cam.5.6.19191 22274713 PMC3277781

[38] Campos LS Leone DP Relvas JB Brakebusch C Fässler R Suter U . Beta1 integrins activate a MAPK signalling pathway in neural stem cells that contributes to their maintenance. Development. (2004) 131:3433–44. doi: 10.1242/dev.01199 15226259

[39] Campos LS . Beta1 integrins and neural stem cells: making sense of the extracellular environment. Bioessays. (2005) 27:698–707. doi: 10.1002/bies.20256 15954093

[40] Leone DP Relvas JB Campos LS Hemmi S Brakebusch C Fässler R . Regulation of neural progenitor proliferation and survival by beta1 integrins. J Cell Sci. (2005) 118:2589–99. doi: 10.1242/jcs.02396 15928047

[41] Bockmayr M Mohme M Klauschen F Winkler B Budczies J Rutkowski S . Subgroup-specific immune and stromal microenvironment in medulloblastoma. Oncoimmunology. (2018) 7:e1462430. doi: 10.1080/2162402X.2018.1462430 30228931 PMC6140816

[42] Riemondy KA Venkataraman S Willard N Nellan A Sanford B Griesinger AM . Neoplastic and immune single-cell transcriptomics define subgroup-specific intra-tumoral heterogeneity of childhood medulloblastoma. Neuro Oncol. (2022) 24:273–86. doi: 10.1093/neuonc/noab135 34077540 PMC8804892

[43] Williamson D Schwalbe EC Hicks D Aldinger KA Lindsey JC Crosier S . Medulloblastoma group 3 and 4 tumors comprise a clinically and biologically significant expression continuum reflecting human cerebellar development. Cell Rep. (2022) 40:111162. doi: 10.1016/j.celrep.2022.111162 35926460 PMC9638015

[44] Lathia JD Mack SC Mulkearns-Hubert EE Valentim CLL Rich JN . Cancer stem cells in glioblastoma. Genes Dev. (2015) 29:1203–17. doi: 10.1101/gad.261982.115 26109046 PMC4495393

[45] Clevers H . The cancer stem cell: premises, promises and challenges. Nat Med. (2011) 17:313–9. doi: 10.1038/nm.2304 21386835

[46] Neftel C Laffy J Filbin MG Hara T Shore ME Rahme GJ . An integrative model of cellular states, plasticity, and genetics for glioblastoma. Cell. (2019) 178:835–849.e21. doi: 10.1016/j.cell.2019.06.024 31327527 PMC6703186

[47] Batlle E Clevers H . Cancer stem cells revisited. Nat Med. (2017) 23:1124–34. doi: 10.1038/nm.4409 28985214

[48] Hemmati HD Nakano I Lazareff JA Masterman-Smith M Geschwind DH Bronner-Fraser M . Cancerous stem cells can arise from pediatric brain tumors. Proc Natl Acad Sci USA. (2003) 100:15178–83. doi: 10.1073/pnas.2036535100 14645703 PMC299944

[49] Singh SK Hawkins C Clarke ID Squire JA Bayani J Hide T . Identification of human brain tumour initiating cells. Nature. (2004) 432:396–401. doi: 10.1038/nature03128 15549107

[50] Suvà ML Rheinbay E Gillespie SM Patel AP Wakimoto H Rabkin SD . Reconstructing and reprogramming the tumor-propagating potential of glioblastoma stem-like cells. Cell. (2014) 157:580–94. doi: 10.1016/j.cell.2014.02.030 24726434 PMC4004670

[51] Soeda A Park M Lee D Mintz A Androutsellis-Theotokis A McKay RD . Hypoxia promotes expansion of the CD133-positive glioma stem cells through activation of HIF-1alpha. Oncogene. (2009) 28:3949–59. doi: 10.1038/onc.2009.252 19718046

[52] Keith B Simon MC . Hypoxia-inducible factors, stem cells, and cancer. Cell. (2007) 129:465–72. doi: 10.1016/j.cell.2007.04.019 17482542 PMC3150586

[53] Semenza GL . Hypoxia-inducible factors in physiology and medicine. Cell. (2012) 148:399–408. doi: 10.1016/j.cell.2012.01.021 22304911 PMC3437543

[54] Nallanthighal S Heiserman JP Cheon D-J . The role of the extracellular matrix in cancer stemness. Front Cell Dev Biol. (2019) 7:86. doi: 10.3389/fcell.2019.00086 31334229 PMC6624409

[55] Hall PE Lathia JD Caldwell MA Ffrench-Constant C . Laminin enhances the growth of human neural stem cells in defined culture media. BMC Neurosci. (2008) 9:71. doi: 10.1186/1471-2202-9-71 18651950 PMC2496909

[56] Danen EH Yamada KM . Fibronectin, integrins, and growth control. J Cell Physiol. (2001) 189:1–13. doi: 10.1002/jcp.1137 11573199

[57] Kazanis I Ffrench-Constant C . Extracellular matrix and the neural stem cell niche. Dev Neurobiol. (2011) 71:1006–17. doi: 10.1002/dneu.20970 21898854 PMC4557210

[58] Staquicini FI Dias-Neto E Li J Snyder EY Sidman RL Pasqualini R . Discovery of a functional protein complex of netrin-4, laminin γ1 chain, and integrin α6β1 in mouse neural stem cells. Proc Natl Acad Sci. (2009) 106:2903–8. doi: 10.1073/pnas.0813286106 19193855 PMC2635839

[59] Fiorilli P Partridge D Staniszewska I Wang JY Grabacka M So K . Integrins mediate adhesion of medulloblastoma cells to tenascin and activate pathways associated with survival and proliferation. Lab Invest. (2008) 88:1143–56. doi: 10.1038/labinvest.2008.89 18794852 PMC2679155

[60] Charles NA Holland EC Gilbertson R Glass R Kettenmann H . The brain tumor microenvironment. Glia. (2012) 60:502–14. doi: 10.1002/glia.21264 22379614

[61] Calabrese C Poppleton H Kocak M Hogg TL Fuller C Hamner B . A perivascular niche for brain tumor stem cells. Cancer Cell. (2007) 11:69–82. doi: 10.1016/j.ccr.2006.11.020 17222791

[62] Engler AJ Sen S Sweeney HL Discher DE . Matrix elasticity directs stem cell lineage specification. Cell. (2006) 126:677–89. doi: 10.1016/j.cell.2006.06.044 16923388

[63] Levental KR Yu H Kass L Lakins JN Egeblad M Erler JT . Matrix crosslinking forces tumor progression by enhancing integrin signaling. Cell. (2009) 139:891–906. doi: 10.1016/j.cell.2009.10.027 19931152 PMC2788004

[64] Paszek MJ Zahir N Johnson KR Lakins JN Rozenberg GI Gefen A . Tensional homeostasis and the Malignant phenotype. Cancer Cell. (2005) 8:241–54. doi: 10.1016/j.ccr.2005.08.010 16169468

[65] Moro L Dolce L Cabodi S Bergatto E Boeri Erba E Smeriglio M . Integrin-induced epidermal growth factor (EGF) receptor activation requires c-Src and p130Cas and leads to phosphorylation of specific EGF receptor tyrosines. J Biol Chem. (2002) 277:9405–14. doi: 10.1074/jbc.M109101200 11756413

[66] Abraham RT . Cell cycle checkpoint signaling through the ATM and ATR kinases. Genes Dev. (2001) 15:2177–96. doi: 10.1101/gad.914401 11544175

[67] Jackson SP Bartek J . The DNA-damage response in human biology and disease. Nature. (2009) 461:1071–8. doi: 10.1038/nature08467 19847258 PMC2906700

[68] Bao S Wu Q McLendon RE Hao Y Shi Q Hjelmeland AB . Glioma stem cells promote radioresistance by preferential activation of the DNA damage response. Nature. (2006) 444:756–60. doi: 10.1038/nature05236 17051156

[69] Diehn M Cho RW Lobo NA Kalisky T Dorie MJ Kulp AN . Association of reactive oxygen species levels and radioresistance in cancer stem cells. Nature. (2009) 458:780–3. doi: 10.1038/nature07733 19194462 PMC2778612

[70] Gray LH Conger AD Ebert M Hornsey S Scott OC . The concentration of oxygen dissolved in tissues at the time of irradiation as a factor in radiotherapy. Br J Radiol. (1953) 26:638–48. doi: 10.1259/0007-1285-26-312-638 13106296

[71] Eke I Dickreuter E Cordes N . Enhanced radiosensitivity of head and neck squamous cell carcinoma cells by β1 integrin inhibition. Radiother Oncol. (2012) 104:235–42. doi: 10.1016/j.radonc.2012.05.009 22748391

[72] Park CC Zhang HJ Yao ES Park CJ Bissell MJ . β1 integrin inhibition dramatically enhances radiotherapy efficacy in human breast cancer xenografts. Cancer Res. (2008) 68:4398–405. doi: 10.1158/0008-5472.CAN-07-6390 18519702 PMC3719863

[73] Storch K Cordes N . Focal adhesion-chromatin linkage controls tumor cell resistance to radio- and chemotherapy. Chemother Res Pract. (2012) 2012:319287. doi: 10.1155/2012/319287 22778951 PMC3385588

[74] Bonnans C Chou J Werb Z . Remodelling the extracellular matrix in development and disease. Nat Rev Mol Cell Biol. (2014) 15:786–801. doi: 10.1038/nrm3904 25415508 PMC4316204

[75] Barcellos-Hoff MH Park C Wright EG . Radiation and the microenvironment - tumorigenesis and therapy. Nat Rev Cancer. (2005) 5:867–75. doi: 10.1038/nrc1735 16327765

[76] Murphy JM Rodriguez YAR Jeong K Ahn E-Y Lim S-T . Targeting focal adhesion kinase in cancer cells and the tumor microenvironment. Exp Mol Med. (2020) 52:877–86. doi: 10.1038/s12276-020-0447-4 32514188 PMC7338452

[77] Dupont S Morsut L Aragona M Enzo E Giulitti S Cordenonsi M . Role of YAP/TAZ in mechanotransduction. Nature. (2011) 474:179–83. doi: 10.1038/nature10137 21654799

[78] Pankov R Yamada KM . Fibronectin at a glance. J Cell Sci. (2002) 115:3861–3. doi: 10.1242/jcs.00059 12244123

[79] Christofides A Strauss L Yeo A Cao C Charest A Boussiotis VA . The complex role of tumor-infiltrating macrophages. Nat Immunol. (2022) 23:1148–56. doi: 10.1038/s41590-022-01267-2 35879449 PMC10754321

[80] Zhang H Ozaki I Mizuta T Matsuhashi S Yoshimura T Hisatomi A . β1-integrin protects hepatoma cells from chemotherapy induced apoptosis via a mitogen-activated protein kinase dependent pathway. Cancer. (2002) 95:896–906. doi: 10.1002/cncr.10751 12209735

[81] Park CC Zhang H Pallavicini M Gray JW Baehner F Park CJ . β1 integrin inhibitory antibody induces apoptosis of breast cancer cells, inhibits growth, and distinguishes Malignant from normal phenotype in three dimensional cultures and *in vivo*. Cancer Res. (2006) 66:1526–35. doi: 10.1158/0008-5472.CAN-05-3071 16452209 PMC2933188

[82] Hu H-H Wang S-Q Shang H-L Lv H-F Chen B-B Gao S-G . Roles and inhibitors of FAK in cancer: current advances and future directions. Front Pharmacol. (2024) 15:1274209. doi: 10.3389/fphar.2024.1274209 38410129 PMC10895298

[83] Sulzmaier FJ Jean C Schlaepfer DD . FAK in cancer: mechanistic findings and clinical applications. Nat Rev Cancer. (2014) 14:598–610. doi: 10.1038/nrc3792 25098269 PMC4365862

[84] Mas-Moruno C Rechenmacher F Kessler H . Cilengitide: the first anti-angiogenic small molecule drug candidate design, synthesis and clinical evaluation. Anti-Cancer Agents Med Chem. (2010) 10:753–68. doi: 10.2174/187152010794728639 21269250 PMC3267166

[85] Stupp R Hegi ME Gorlia T Erridge SC Perry J Hong Y-K . Cilengitide combined with standard treatment for patients with newly diagnosed glioblastoma with methylated MGMT promoter (CENTRIC EORTC 26071-22072 study): a multicentre, randomised, open-label, phase 3 trial. Lancet Oncol. (2014) 15:1100–8. doi: 10.1016/S1470-2045(14)70379-1 25163906

[86] Echavidre W Picco V Faraggi M Montemagno C . Integrin-αvβ3 as a therapeutic target in glioblastoma: Back to the future? Pharmaceutics. (2022) 14:1053. doi: 10.3390/pharmaceutics14051053 35631639 PMC9144720

